# Solvent-free and time-efficient Suzuki–Miyaura reaction in a ball mill: the solid reagent system KF–Al_2_O_3_ under inspection

**DOI:** 10.3762/bjoc.6.7

**Published:** 2010-01-22

**Authors:** Franziska Bernhardt, Ronald Trotzki, Tony Szuppa, Achim Stolle, Bernd Ondruschka

**Affiliations:** 1 Institute for Technical Chemistry and Environmental Chemistry, Friedrich-Schiller University Jena, Lessingstraße 12, D-07743 Jena, Germany

**Keywords:** ball milling, C–C coupling, KF–Al_2_O_3_, palladium, solid reagent system

## Abstract

Although a plethora of synthetic procedures mediated by KF-loaded aluminas is available in the literature, there is almost no data concerning the influence of parameters such as alumina modification or KF-loading on experimental results. Hence, the Pd-catalyzed, solvent-free Suzuki–Miyaura reaction was chosen as model reaction to investigate the effect of the above mentioned parameters on the results of coupling reactions. The results from ball milling experiments led to the conclusion that self-prepared and commercially available KF–Al_2_O_3_ differ in water content. The higher the residual water content, the higher are the product yields.

## Introduction

Over the last two decades, a major trend in the field of organic chemistry has been evident: environmentally friendly processes using safer reagents, generating fewer side products and requiring less use of solvents are in vogue [1–2]. This trend has led to an explosive growth in the fields of solvent-free synthesis [3–13] and solid-supported reagents [14–18] such as potassium fluoride on alumina (KF–Al_2_O_3_) [19–20]. This versatile reagent was originally introduced in 1979 by Ando et al. as a useful agent for inducing alkylation reactions [21]. Additional benefits have been derived by taking advantage of the strongly basic nature of KF–Al_2_O_3_, which can replace organic bases in a number of reactions including, but not limited to, alkylations or epoxidations [18–20,22–29]. Apart from the plethora of synthetic applications, there is one major issue that has not been addressed in the literature so far. The experimental procedures for the preparation of the KF–Al_2_O_3_ reagents often differ significantly from each other, as exemplified for the most recent procedures summarized in Table 1 [21,30–34]. Besides information on the synthesis itself, data on the type of alumina used for preparation is lacking and only a few authors have been interested in the issue of alumina modification and to what extent synthesis and modification influence the reactions involving KF-loaded aluminas [21,35].

**Table 1:** Representative experimental procedures for the preparation of KF–Al_2_O_3_.

Reference^a^	Preparation sequence

[21]	i) suspension in water
	ii) removal of water in vacuum (50–60 °C)
	iii) drying at 75/150 °C
[30]	i) suspension in water
	ii) filtration and “sucked to free flowing powder”
[31–32]	i) suspension in water
	ii) removal of water in vacuum (100 °C)
	iii) removal of residual water with ethanol
	iv) drying at 110 °C
[33]	i) grinding of commercially available KF–Al_2_O_3_
	ii) “exposed to air […] to allow for hydration of the surface”
[34]	i) suspension in THF
	ii) removal of THF in vacuum
	iii) drying at 250 °C (0.7 mbar)

^a^Only the oldest reference is given.

The investigations presented in this study focused on the preparation of KF–aluminas with respect to preparation and storage conditions, KF-loading, and the use of alumina modification. The as-prepared solid reagent systems (**SRS**) were then applied in solvent-free Pd-catalyzed Suzuki–Miyaura cross-coupling reactions of aryl bromides with phenylboronic acid, and the performances of the individual **SRS** were compared [33–34,36–42].

## Results and Discussion

**Scheme 1:**
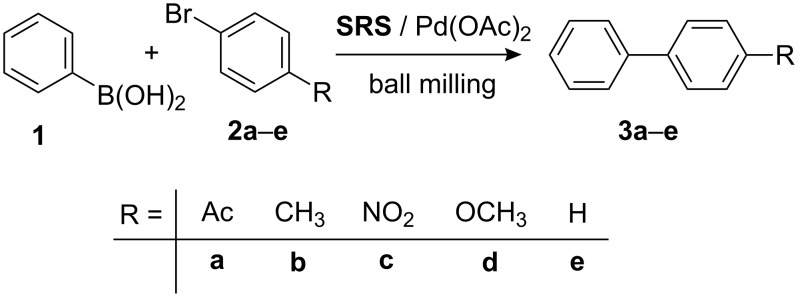
Suzuki–Miyaura reaction of phenylboronic acid (**1**) with aryl bromides **2** yielding substituted biaryls **3** under ball milling conditions (palladium-catalyzed and **SRS**-assisted).

Incorporation of KF–Al_2_O_3_ as a basic component in organic synthesis is a wide research field [18–29]. However, the application of this reagent is rather problematic, as different reaction protocols for the synthesis of reagents have been published (Table 1) [21,30–34], which differ in the type of alumina used for synthesis and KF-loading. Especially the basicity and the modification of alumina are often not given, which leads to reproducibility problems of experimental procedures. Herein three different types of aluminas (Table 2) have been applied in the synthesis of KF–aluminas, all denoted generally as **SRS**. In the case of KF-loaded aluminas types **SRS1a–3a,** the last number indicates the KF content by weight. For comparison, two commercially available KF–aluminas (**SRS4a** and **SRS5a**) were used in this study. Table 2 summarizes the basic characteristics of the **SRS**. The denotation of the modification of Al_2_O_3_ in Table 2 resulted from XRD analyses (cf. Supporting Information File 1) and specific surface areas *A*_S_ were determined using 6-point-BET measurements. γ-Al_2_O_3_ **SRS1** and **SRS3** differ in their surface characteristics. **SRS1** possesses a neutral surface, whereas **SRS3** shows a higher basicity that was verified by pH measurements of aqueous suspension of the pure aluminas. Investigating the influence of different **SRS**, the Pd(OAc)_2_-assisted Suzuki–Miyaura coupling of phenylboronic acid (**1**) with different aryl bromides **2** furnishing *p*-substituted biphenyls **3** (Scheme 1) was chosen. Reactions were performed in a mechanical manner by co-grinding the reactants with agate milling balls using a planetary ball mill as the source for alternative energy input [4–6,9,36,40–56]. It is worth mentioning that all results presented in this paper were achieved by comminuting the reactants for just 10 min [40–41] without the presence of any additional stabilizing or activating ligands. Compared to the original published procedure for the Suzuki–Miyaura reaction [36] or the protocols featured by Mack and Frejd and their coworkers for Sonogashira [54] and Heck–Jeffery couplings applying ball milling conditions [55–56], respectively, the reaction times were substantially reduced from several hours to a few minutes following the present reaction protocol.

**Table 2:** Characteristics of the base components applied in Suzuki–Miyaura reactions as **SRS**.

Denotation	Composition^a^	*A*_S_ [m^2^ g^−1^]^b^

**SRS1**	γ-Al_2_O_3_	148
**SRS2**	α-Al_2_O_3_	114
**SRS3**	γ-Al_2_O_3_	185

**SRS1a-32**	**SRS1** + 32 wt % KF	42
**SRS2a-32**	**SRS2** + 32 wt % KF	34
**SRS3a-32**	**SRS3** + 32 wt % KF	44
**SRS4a**^c^	KF–Al_2_O_3_ (32 wt % KF)	32
**SRS5a**^c^	KF–Al_2_O_3_ (40 wt % KF)	ND

^a^Modification deduced from XRD measurements. Preparation of the KF-loaded aluminas by wet-impregnation method. ^b^Using 6-point-BET method. ^c^Commercially available form.

**Figure 1:**
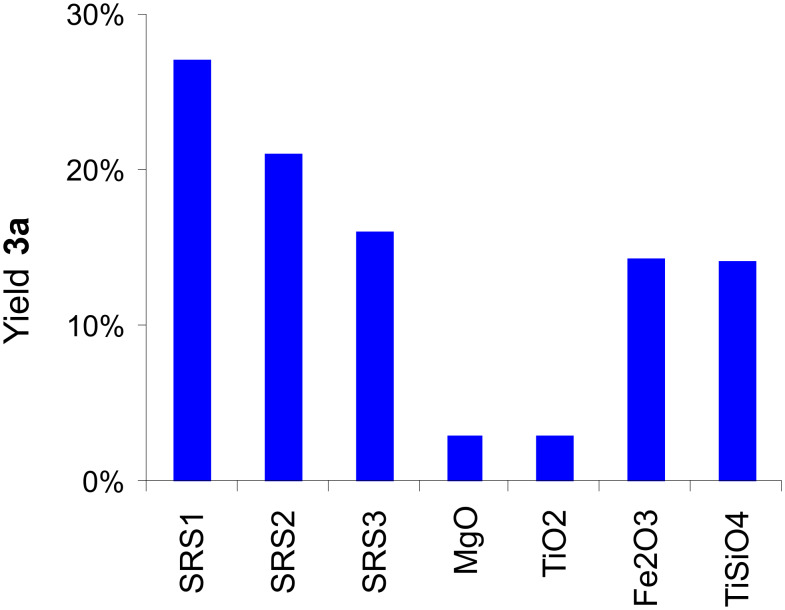
Results of the Suzuki–Miyaura reaction of phenylboronic acid (**1**) with *p*-bromoacetophenone (**2a**; cf. Scheme 1) assisted by various metal oxides [5 g; **SRS1**–**3**: cf. Table 2; ball milling: 2 agate milling beakers (*V* = 45 ml), 6 agate milling balls (*d* = 15 mm) per beaker, 800 rpm, 10 min; batch: 5 mmol **2a**, 124 mol % **1**, 3.6 mol % Pd(OAc)_2_].

**Figure 2:**
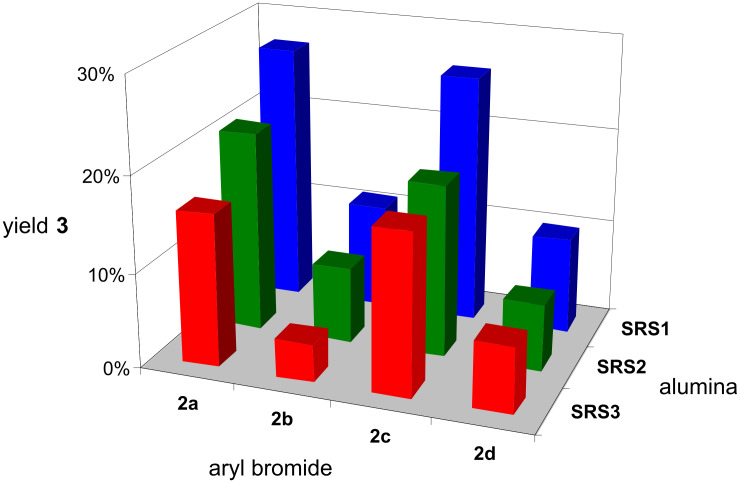
Results of the Suzuki–Miyaura reaction according to Scheme 1 assisted by pure aluminas [5 g **SRS1**–**3**: cf. Table 2; ball milling: 2 agate milling beakers (*V* = 45 ml), 6 agate milling balls (*d* = 15 mm) per beaker, 800 rpm, 10 min; batch: 5 mmol aryl bromide, 124 mol % phenylboronic acid, 3.6 mol % Pd(OAc)_2_].

A comparison of the results from the Suzuki–Miyaura coupling of **1** with *p*-bromoacetophenone (**2a**) assisted by pure aluminas **SRS1–3** with the results of experiments performed in the presence of other binary oxides (MgO, SiO_2_, TiO_2_, CeO_2_, Fe_2_O_3_) is presented in Figure 1. The results strongly indicate that the inherent basicity of the aluminas is beneficial for the reaction, although amphoteric oxide, Fe_2_O_3_, and ternary titanium silicalite, TiSiO_4_, showed moderate conversions in the range of basic γ-Al_2_O_3_ **SRS3**. Contrary to microwave-assisted reactions, MgO showed only little conversion when used as a base under ball milling conditions [34], whereas neutral oxides SiO_2_ and CeO_2_ did not show any conversion. Since the results in Figure 1 are restricted to one aryl bromide (**2a**), experiments were expanded by taking into account the ability of the substituents to influence the experimental results. Figure 2 shows the results of blank tests regarding the base component, in which the C–C coupling was carried out in the presence of pure aluminas **SRS1–3** without the addition of any further basic component. The experiments resulted in the following conclusion: it is in principle possible to use pure Al_2_O_3_ as “base component” without any modification such as the addition of KF or any other basic additive [33–34,36–42,57–58]. The yield of the coupling product depends on the reactivity of the aryl bromide and the reverse basicity of the **SRS** used. **SRS1** (neutral γ-Al_2_O_3_) yielded the best results with the tested aryl bromides, followed by basic **SRS2** (α-Al_2_O_3_) and **SRS3** (γ-Al_2_O_3_). The preference of neutral to basic aluminas has been recently shown in three-component reactions affording thiochromeno[2,3-*b*]pyridine derivatives [24]. The results were in clear contrast to that of the microwave-assisted Pd(PPh_3_)_4_-catalyzed solid-state Suzuki–Miyaura reaction protocol presented by Saha et al., revealing that basic alumina yields the best results, whereas neutral alumina is inactive [35]. The authors assumed that alumina with basic surface characteristics allows the in situ formation of “interfacial” boronic esters resulting from the electronic interaction between electron-deficient boron in boronic acid and surface oxygen of alumina (ArB(OH)_2_–O–Al(O–)_3_). The reported results also confute the results from microwave-assisted cross-coupling with pure basic alumina, which was seen to be completely inert for this type of reaction [33]. Apparently co-grinding of all reactants in a ball mill leads to in situ activation, which enhances the capability of aluminas to act as a base in Suzuki–Miyaura reactions probably because of the generation of new highly active surfaces [59] including the defect sites that allow the activation of the boronic acid as mentioned above.

Besides the intriguing fact that Al_2_O_3_ was shown to act solely as a base in the investigated cross-coupling to biphenyls, the resulting yields were inferior to the yield required for application in synthesis. To increase the basicity of the **SRS** KF-loaded aluminas, **SRS1a–3a**, which were promising for application in the Suzuki–Miyaura reaction [33–34,36–42] were prepared. In combination with alumina, KF seemed to be the best choice, as the reactions with other inorganic bases (for example KOH, NaF, Na_2_CO_3_) resulted in lower conversion for microwave-assisted reaction protocols [33–34]. A few literature studies report different experimental procedures for the preparation of the **SRS** KF–Al_2_O_3_, which mainly differ in the way water is removed after the synthesis of the reagent [21,30,33,39,60]; see Table 1. Due to these different reaction protocols, investigations focused on the conditions that prevailed during the synthesis and storage of the KF–aluminas. Table 3 summarizes the endeavors leading to the conclusion that the use of water as solvent for the preparation of **SRS** results in more active reagent systems [33,39–42], than, for instance, THF [34,60]. The preference of water as solvent in the preparation step was also demonstrated in the hydrolysis of warfare agents, whereas the synthesis of KF–Al_2_O_3_ with methanol or ethanol resulted in decreased reaction rates [29]. Additionally, the data compiled in Table 3 reveal that removal of the solvent by evaporation in vacuum and storage of the as-prepared **SRS** either in air atmosphere or in a desiccator over KOH are possible. Removal of residual water by calcination for 2 h at 300 °C afforded a strongly deactivated material, confirming the earlier studies on the application of calcinated KF–Al_2_O_3_ in the O-methylation of phenols [21]. For obtaining these results, materials were stored in a desiccator over KOH and vacuum for further applications to avoid uncontrollable uptake of water as indicated in the procedures of Kabalka et al. (“exposed to air over a period of days to allow for hydration of the surface”) [33] as well as for preventing the material from incorporation of CO_2_ from air (formation of K_2_CO_3_) [30,61]. 

**Table 3:** Influence of preparation and storage conditions of KF-loaded aluminas on the yield of biphenyl (**3e**) in the Suzuki–Miyaura reaction^a^ of phenylboronic acid (**1**) with bromobenzene (**2e**; Scheme 1).

		Yield of **3e** (%) for preparation^b^/storage conditions		
KF-loaded Al_2_O_3_^c^	Solvent^d^	Air atmosphere	Vacuum/KOH	Calcination^e^

**SRS3a-20**^f^	THF	52	61	26
**SRS3a-20**^f^	water	68	92	35
**SRS3a-40**	water	95	98	50
**SRS1a-40**	water	97	98	54

^a^Ball milling: 2 agate milling beakers (*V* = 45 ml), 7 agate milling balls (*d* = 15 mm) per beaker, 800 rpm, 10 min; batch: 5 g KF-loaded Al_2_O_3_, 5 mmol **2e**, 124 mol % **1**, 3.6 mol % Pd(OAc)_2_. ^b^For general preparation sequence see experimental section. ^c^Denotation cf. Table 2; the number after the hyphen indicates the KF content of the **SRS** in wt %. ^d^Solvent used for preparation of **SRS**. ^e^2 h at 300 °C and afterwards storage in desiccator over KOH (N_2_). ^f^2.2 mol % Pd(OAc)_2_.

The next feature, which is often changed in a non-systematic manner when working with KF–Al_2_O_3_, is the loading of the aluminas. Only in a few examples, this parameter was investigated by preparing and applying different materials varying in their KF-loadings [27,40,62–63]. Thus, different supports with KF concentrations ranging from 10–50 wt % were prepared in order to investigate the influence of the KF content on the Suzuki–Miyaura reaction of **1** with **2a** and with bromobenzene (**2e**; Scheme 1) [40]. The yields that resulted after ball milling are shown in Table 4, which reveals that low concentrations of KF afforded poor yields of coupling product **3**. Very high yields were obtained for KF-loaded aluminas with more than 20 wt % KF. This general trend is in accordance with the other reactions applying KF–Al_2_O_3_ as either stoichiometric or catalytic reagent: transesterification of vegetable oils [27], hydrolysis reactions [29], O-methylation of phenol [62], and Michael reactions [63]. In conclusion, it appears that a KF content of 30–40 wt % is sufficient for a mechanochemically initiated conversion of **1** and **2** yielding **3**.

**Table 4:** Effect of KF concentration in case of **SRS1a** on the yield of the Suzuki–Miyaura reaction^a^ of phenylboronic acid (**1**) with *p*-bromoacetophenone (**2a**) and with bromobenzene (**2e**; Scheme 1).

	Yield (%)	
KF-loading (wt %)	**3a**	**3e**

10	42	77
20	94	94
30	97	95
32	94	98
32 (**SRS4a**)^b^	55	ND
40	94	>99
40 (**SRS5a**)^b^	51	85
50	96	>99

^a^Ball milling: 2 agate milling beakers (*V* = 45 ml), 6 agate milling balls (*d* = 15 mm) per beaker, 800 rpm, 10 min; batch: 5 g **SRS1a**, 5 mmol **2**, 124 mol % **1**, 3.6 mol % Pd(OAc)_2_. ^b^Commercially available form.

**Figure 3:**
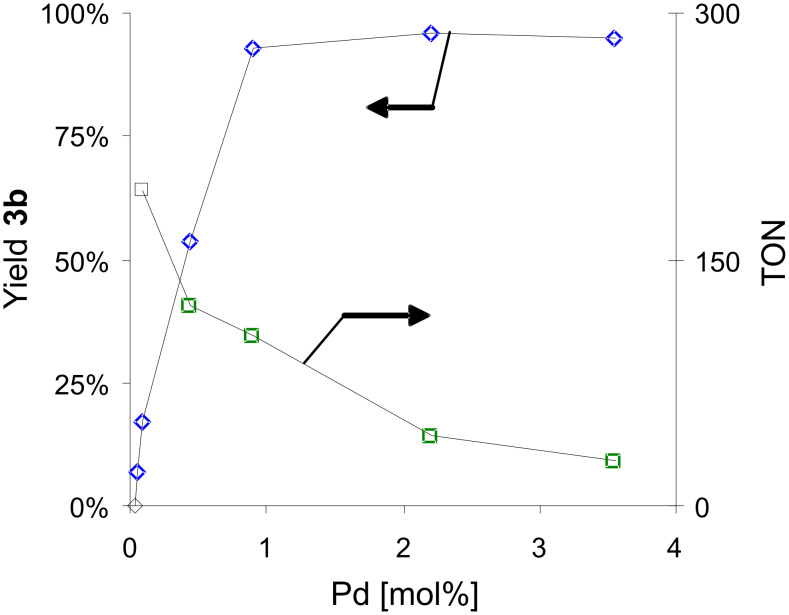
Influence of Pd(OAc)_2_ concentration on the results of the Suzuki–Miyaura reaction of phenylboronic acid (**1**; 124 mol %) with *p*-bromotoluene (**2b**; 5 mmol) assisted by **SRS1a-40** [5 g: cf. Table 2; ball milling: 2 agate milling beakers (*V* = 45 ml), 7 agate milling balls (*d* = 15 mm) per beaker, 800 rpm, 10 min].

Reproducing the experimental protocol of Basu et al., the experiments listed in Table 3 (THF for the preparation of **SRS3a-20**) [34,60] were performed using 2.2 mol % Pd instead of 3.6 mol % as for the other experiments. The investigation of the influence of Pd concentration on the yield of 4-methylbiphenyl (**3b**) assisted by **SRS1a-40** revealed that Pd-loadings higher than 1 mol % led to satisfactory yields of coupling product (Figure 3). The application of lower Pd concentrations (< 2 µmol) under these experimental prerequisites afforded moderate conversions only, foreshadowing that reactions with ultra-low amounts of Pd [64–66] required either longer reaction times or co-grinding with higher-weight milling balls (ZrO_2_, stainless steel, tungsten carbide) [40,67–68]. 

**Figure 4:**
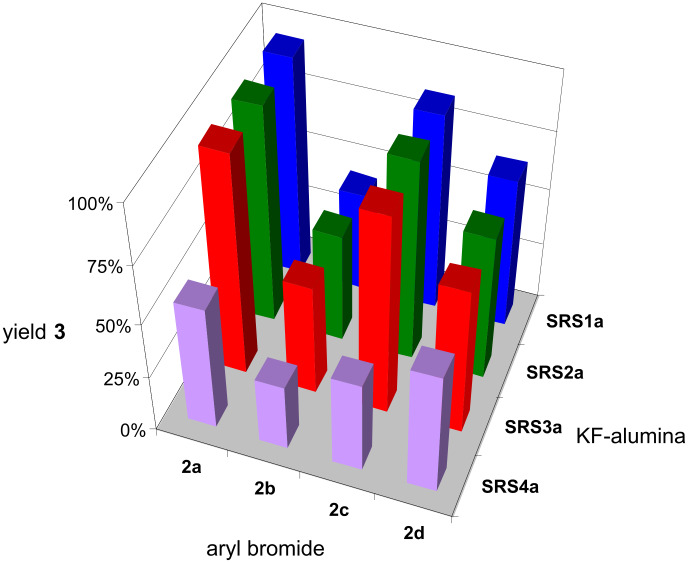
Results of the Suzuki–Miyaura reaction according to Scheme 1 assisted by KF-loaded aluminas [5 g **SRS1a**–**4a**, 32 wt % KF: cf. Table 2; ball milling: 2 agate milling beakers (*V* = 45 ml), 6 agate milling balls (*d* = 15 mm) per beaker, 800 rpm, 10 min; batch: 5 mmol aryl bromide, 124 mol % phenylboronic acid, 3.6 mol % Pd(OAc)_2_].

The application of the as-prepared KF-loaded **SRS** in the other Suzuki–Miyaura reactions led to interesting results summarized in Figure 4. The results with KF–Al_2_O_3_ **SRS1a**–**4a** confirm the statement that the yield of the desired coupling product depends on the educt and on the influence of its substituents on the reaction course. However, compared to the reactions performed with pure aluminas (Figure 2), a significant increase in the yield of coupling products **3** was observed while using KF-loaded aluminas, clearly indicating that a base is necessary for a successful C–C coupling reaction [33–34,36–42,57–58]. The purchased KF–Al_2_O_3_ (**SRS4a**) system shows the worst results for all tested aryl bromides, except for **2b** (**SRS2a**). The self-made supports **SRS1a**–**3a** show comparable results for each tested aryl bromide, except **2b**. 

**Figure 5:**
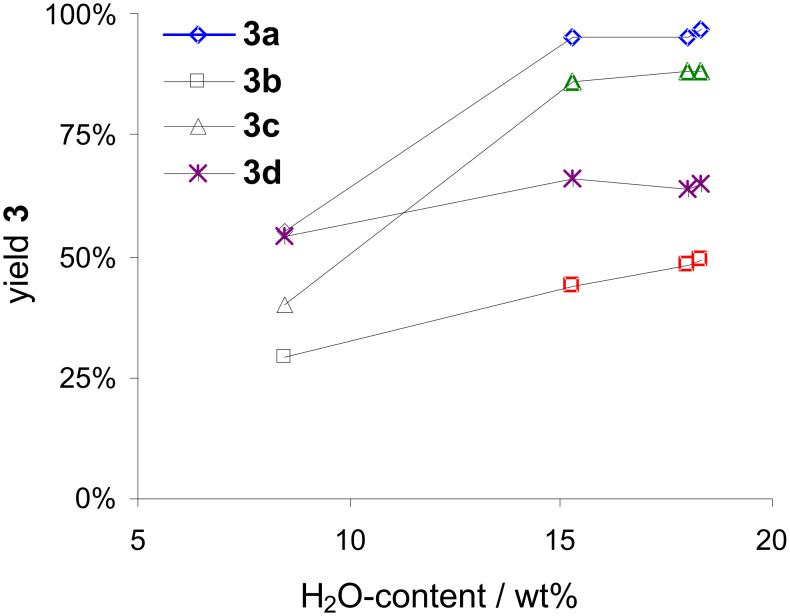
Dependence of the yield of Suzuki–Miyaura cross-coupling product (Scheme 1) from water content (Karl-Fischer titration) of KF-loaded aluminas [5 g **SRS1a–4a**, 32 wt % KF: cf. Table 2; ball milling: 2 agate milling beakers (*V* = 45 ml), 6 agate milling balls (*d* = 15 mm) per beaker, 800 rpm, 10 min; batch: 5 mmol aryl bromide, 124 mol % phenylboronic acid, 3.6 mol % Pd(OAc)_2_].

However, the results in Figure 4 are difficult to interpret regarding the influence of the different types of **SRS** used. The type of modification and the basicity of the initially applied aluminas (**SRS1**–**3**; cf. Table 2) are not as they have been expected to be. The KF-loaded aluminas differed in their residual water content (determined by Karl–Fischer-titration), because of the preparation of aluminas with KF via wet-impregnation using deionized water as solvent, which was 13.4, 18.0, 19.3, and 8.5 wt % for **SRS1a**, **SRS2a**, **SRS3a**, and **SRS4a** (32 wt % KF), respectively [69]. Comparing these values with the yields of coupling products reveals a strong dependency: an increase in the water content leads to an increase in the yields of the desired products (Figure 5). According to these results, the Suzuki–Miyaura reaction depends on the solvent, even though the reaction can be performed without any solvent (solventless conditions). This observation is in accordance with other ball milling or solvent free protocols, thus revealing that the addition of small portions of water is beneficial for the reaction [29,70]. From the data in Figure 5, another interesting fact can be recognized: the strength of the influence of residual water seems to be also dependent on the polarity of the applied aryl bromide. The higher the polarity of the aryl halide, the higher is the influence of the residual water content on the product yield. *p*-Bromoacetophenone (**2a**) and *p*-bromonitrobenzene (**2c**) have a log *P*_oct/wat_-coefficient of 2.43 ± 0.31 and 2.55 ± 0.30, respectively, accounting for a higher solubility in water, than **2b** (log *P*_oct/wat_ = 3.45 ± 0.28) and *p*-bromoanisole (**2d**; log *P*_oct/wat_ = 3.17 ± 0.39) [71]. 

Thermoanalyses (TGA, DTA) of the as-prepared KF-loaded aluminas strongly indicated a mass loss in the range of 50–150 °C, accompanied by an exothermic reaction typical for a dehydration reaction (cf. Supporting Information File 1). This accounts for the fact that water in the present case is only physisorbed. The results are in clear contrast to the studies of Verziu et al., which report a significant mass loss at 300 °C combined with an endothermic signal in the DTA [27]. The observed water release is assumed to result from the condensation of AlO(OH) species (Boehmite) to Al_2_O_3_ on the surface [72]. However, the authors were not able to prove the change in alumina modification as the XRD results only point out the formation of K_3_AlF_6_ phases, but not the proposed phase transformation. The main difference between our present study and the recently published study is the fact that, according to the previous study, the formation of K_3_AlF_6_ and KOH (Equation 1) [30,61,73–74] was reported to take place during calcination, whereas, in our study, this reaction occurs already in the solution. This was indicated by XRD measurements of the freshly prepared KF-loaded aluminas (cf. Supporting Information File 1).

[1]
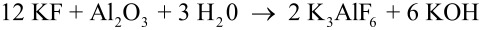


Investigating the influence of water content, some further experiments were conducted in order to (1) activate **SRS** with low water content by the addition of water and (2) deactivate self-prepared supports by calcination at elevated temperatures. **SRS2b** was prepared by the calcination of **SRS2a** at 300 °C for 2 h, which resulted in a decrease of the water content from 18.0 wt % to 5 wt % for **SRS2b**. When this material was applied to the Suzuki–Miyaura reaction, a significant decrease in the yield of coupling product **3a** was observed (Table 5). Results pointing in the same direction were achieved when the KF-loaded aluminas were calcinated directly after removal of the solvent in the preparation step (Table 3). ^19^F-MAS NMR studies indicate that calcination at *T* > 300 °C afforded decomposition of [AlF_6_]^3−^-substructures decreasing the catalytic activity, whereas in case of “gentle” drying at 100 °C, the activity remained unaffected [61]. On the other hand, suspending **SRS4a** and **SRS5a** in deionized water, removal of the solvent in vacuum (similar to the preparation of **SRS1a–3a**), and the application of the hydrated KF-loaded aluminas **SRS4b** and **SRS5b** to the synthesis afforded the coupling product in increased amounts compared to the untreated **SRS** (Table 5). These results support the hypothesis that the performance of the reaction is directly connected to the residual water content of the applied **SRS**. 

**Table 5:** Influence of different solid reaction systems (**SRS**) on the yield of the Suzuki–Miyaura reaction^a^ of phenylboronic acid (**1**) with *p*-bromoacetophenone (**2a**; Scheme 1).

	Preparation	Solvent^b^	Yield **3a** (%)

**SRS2a-32**	32 wt % KF on α-Al_2_O_3_ by wet impregnation	water	92
**SRS2b-32**	calcination of **SRS2a**^c^	–	44

**SRS3a-20**	20 wt % KF on γ-Al_2_O_3_ by wet impregnation + calcination^c^	THF	25^d^
**SRS3a-20**	addition of 100 µl water to standard batch size	–	27^d^

**SRS4a-32**	commercially available	–	48
**SRS4b-32**	hydration of **SRS4a**^e^	water	59

**SRS5a-40**	commercially available	–	51
**SRS5b-40**	hydration of **SRS5a**^e^	water	73

^a^Ball milling: 2 agate milling beakers (*V* = 45 ml), 6 agate milling balls (*d* = 15 mm) per beaker, 800 rpm, 10 min; batch: 5 g **SRS**, 5 mmol **2a**, 124 mol % **1**, 3.6 mol % Pd(OAc)_2_. ^b^Solvent used for preparation of **SRS**. ^c^For 2 h at 300 °C (cf. experimental section). ^d^Agate milling balls (7 ×15 mm) per beaker; 2.2 mol % Pd(OAc)_2_. ^e^For more details see experimental section.

## Conclusion

The influence of the type of alumina precursor used for the preparation of KF-loaded aluminas as basic solid reagent systems (**SRS)** for the solvent free Suzuki–Miyaura coupling of aryl bromides with phenylboronic acid induced by ball milling was investigated. Coupling experiments with unloaded aluminas revealed that reactivity follows the inverse basicity of KF free **SRS** applied for synthesis. Loading of the aluminas with KF in different concentrations leads to the conclusion that the outcome of the reaction is independent of the KF content at a concentration of more than 20 wt % KF. In comparison to the reaction with pure aluminas, KF-loaded **SRS** showed a significantly increased activity in the Suzuki–Miyaura reaction under ball milling conditions. In addition to the reactivity of the substrates (substituent effects), the activity of the as-prepared KF–Al_2_O_3_ strongly depends on the residual water content. The extent of water influence on the performance of the coupling reaction with different aryl bromides seems to be in accordance with their polarity.

As a major conclusion, the results presented here reveal that ball milling is a reaction tool affected by several variables. Besides parameters evidently influencing the reactions (for example catalyst loading, reaction time, and rotation frequency), the influence of other parameters (for example basicity of the filling material, water content of reactants) on the results of the reactions becomes clear only on closer inspection.

## Experimental

**General remarks:** All reagents were purchased from commercial suppliers and used without further purification. **SRS4a** and **SRS5a** were purchased from Sigma-Aldrich (cf. Supporting Information File 1) and used without further pre-treatment. Ball milling was conducted using the planetary ball mill “Pulverisette 7 classic line” (Fritsch GmbH, Germany). For balancing, two grinding beakers (*V* = 45 ml) of nearly the same weight were placed inside the ball mill. The purity of all the compounds was checked using capillary gas chromatography. Analyses of the reaction mixtures were carried out by GC-FID and GC-MSD. Conditions GC-FID: HP 5, 30 m × 0.32 mm × 0.25 µm, H_2_ – 12 psi, program: 50 °C (hold for 3 min), 30 K min^−1^ up to 280 °C (hold for 5 min), injector temperature: 280 °C, detector temperature: 300 °C. Conditions GC-MSD: HP 5, 30 m × 0.32 mm × 0.25 µm, He – 12 psi, program: 50 °C (hold for 3 min), 30 K min^−1^ up to 280 °C (hold for 5 min), injector temperature: 280 °C, detector: EI. All product yields reported in this study were determined by GC-FID and are comparable with the isolated ones. The reported yields were corrected by means of different FID-sensitivity for the substrates and products.

**Water content of the solid SRS:** 0.1 g of the **SRS** was suspended in methanol (4 ml, water-free) and sonicated for 30 min. After equilibration (24 h), the measurements were performed at room temperature using a Karl–Fischer Titrator “Aqua 3000”. Values were corrected by blank value of the solvent.

**Standard preparation of the KF-loaded SRS (SRS1a–3a):** In a 250 ml Erlenmeyer flask, potassium fluoride (0.275 mol, 16 g) was dissolved in deionized water (25 ml). Subsequently, alumina (**SRS1–3**; cf. Table 2; 0.333 mol, 34 g) and more deionized water (25 ml) were added to the solution under slow stirring. Stirring was maintained for 1 h. The solvent was removed by evaporation in vacuum (40 mbar, 60 °C) and stored in a desiccator over potassium hydroxide (batch size for 50 g KF–Al_2_O_3_ with a KF content of 32 wt %). The preparation of the other support materials followed the same procedure with different KF/alumina ratios.

**Preparation of SRS2b:** The as-prepared **SRS2a** (10 g) was calcinated in air-atmosphere for 2 h at 300 °C using the microwave oven MLS 1200 Pyro (MLS GmbH, Germany). The resulting dehydrated KF-loaded alumina was stored in a desiccator over potassium hydroxide.

**Preparation of SRS4b and SRS5b:** In a 250 ml Erlenmeyer flask, 50 g of the as-received KF-loaded alumina **SRS4a** or **SRS5a** (32 wt % or 40 wt % KF, respectively) was dissolved in deionized water (50 ml) and was stirred for at least 1.5 h. The solvent was removed by evaporation in vacuum (40 mbar, 60 °C) and stored in a desiccator over potassium hydroxide.

**Typical experimental procedure:** **SRS** (5 g), aryl bromides (**2a–e**; 5 mmol), phenylboronic acid (**1**; 6.19 mmol, 0.755 g, 124 mol %), Pd(OAc)_2_ (0.18 mmol, 0.04 g, 3.56 mol %) were added to the grinding beaker (agate, *V* = 45 ml) already equipped with the milling balls (6× agate, *d* = 15 mm) and placed inside the planetary ball mill Pulverisette 7 (Fritsch GmbH, Germany). Another grinding beaker filled with a similar batch was mounted on the opposite position of the rotating disc. The mixtures were subsequently milled at 800 rpm for 10 min. The crude product was quenched immediately using 2 ml of deionized water and extracted using 3 ml of the respective solvent (**2a**,**c**: ethyl acetate; **2b**,**d,e**: *tert*-butylmethylether). A sample of the organic phase was analyzed using GC-FID.

## Supporting Information

File 1 Source data of the used aluminas **SRS1–3** and KF-loaded aluminas (**SRS4a**, **SRS5a**), thermal analyses data (TGA, DTA) and XRD-spectra from the freshly prepared KF-loaded aluminas **SRS1a–3a** and the respective data for purchased **SRS4a**
