# Supplementary material for: Solvent-free and time-efficient Suzuki–Miyaura reaction in a ball mill: the solid reagent system KF–Al2O3 under inspection
Source: Beilstein J Org Chem. 2010 Jan 22;6:7. doi: 10.3762/bjoc.6.7 (PMC2870998; doi:10.3762/bjoc.6.7)
Supplement: File 1 — Source data of the used aluminas SRS1–3 and KF-loaded aluminas (SRS4a, SRS5a), thermal analyses data (TGA, DTA) and XRD-spectra from the freshly prepared KF-loaded aluminas SRS1a–3a and the respective data for purchased SRS4a [file Beilstein_J_Org_Chem-06-07-s001.pdf]

Supporting Information:

Solvent-free and time-efficient Suzuki–Miyaura reaction in a ball mill: the solid reagent system KF–Al_2O_3 under inspection

Franziska Bernhardt, Ronald Trotzki, Tony Szuppa, Achim Stolle* and Bernd Ondruschka

Address: Institute for Technical Chemistry and Environmental Chemistry, Friedrich-Schiller University Jena, Lessingstraße 12, D-07743 Jena, Germany

Fax: +49(0)3647948402; Tel: +49(0)3647948413

Email: Achim Stolle - Achim.Stolle@uni-jena.de

*Corresponding author

Table of Contents

Source data of the used aluminas and KF-loaded aluminas 3

Thermal analyses of the freshly prepared KF-loaded aluminas 4

XRD measurements of the freshly prepared KF-loaded aluminas 7

Acknowledgment 7

Source data of the used aluminas and KF loaded aluminas

(Denotation cf. Table 2 of the manuscript)

Particle size distribution was measured using a LS 13320 Particle Size Analyzer (Beckman Coulter Inc., USA). The listed particle sizes represent *d* at maximal intensity matching the d_{90}-values with good proximity.

Water content of the solid SRS: 0.1 g of the **SRS** was suspended in methanol (4 ml, water-free) and sonicated for 30 min. After equilibration (24 h), measurements were performed at room temperature using a Karl–Fischer Titrator "Aqua 3000". Values were corrected by blank value of the solvent.

X-ray diffraction (XRD) experiments were performed on a Kristalloflex D5000 diffractometer (Siemens, Germany) with CuK_{α} radiation (E = 37 keV; I = 40 mA) at room temperature using a Ni filter to identify the crystalline phases of the untreated and ball milled aluminas.

SRS1: alumina (90 active, neutral, for column chromatography) from Merck; *d*: 88 µm (producer: 63–200 µm); water content: 2%, modification: γ-Al_2O_3

SRS2: alumina (150 basic, type T) from Merck; *d*: 154 µm (producer: 63–300 µm); water content: 3%, modification: α-Al_2O_3

SRS3: alumina (basic) from Fluka; *d*: 106 µm (producer: 50–150 µm); water content: 2%, modification: γ-Al_2O_3 (with minor impurities of α-Al_2O_3)

SRS4a: KF loaded alumina (32 wt % KF) from Fluka; *d*: 116 µm; water content: 8%

SRS5a: KF loaded alumina (40 wt % KF) from Aldrich; *d*: not determined; water content: 7%

Thermal analyses of the freshly prepared KF loaded aluminas

Thermal analyses (TGA and DTA) were performed using the Thermogravimetric Analyzer TGA-50 (Shimadzu, Japan) with Pt cell under inert reaction atmosphere (N_2). The following temperature program was used for analyses: 10 K min^{-1} up to 100 °C; 20 K min^{-1} up to 1000 °C.

The following TGA and DTA-curves (Figures S1-4) result from measurements of the freshly prepared KF loaded aluminas **SRS1a–3a** (32 wt % KF; cf. Table 2 of the manuscript). The KF–Al_2O_3 system **SRS4a** (32 wt % KF) was purchased from Sigma–Aldrich and analyzed as-received without any pretreatment. Storage of the materials in a desiccator over potassium hydroxide over a period of six months revealed no significant changes in the TGA and DTA curves.

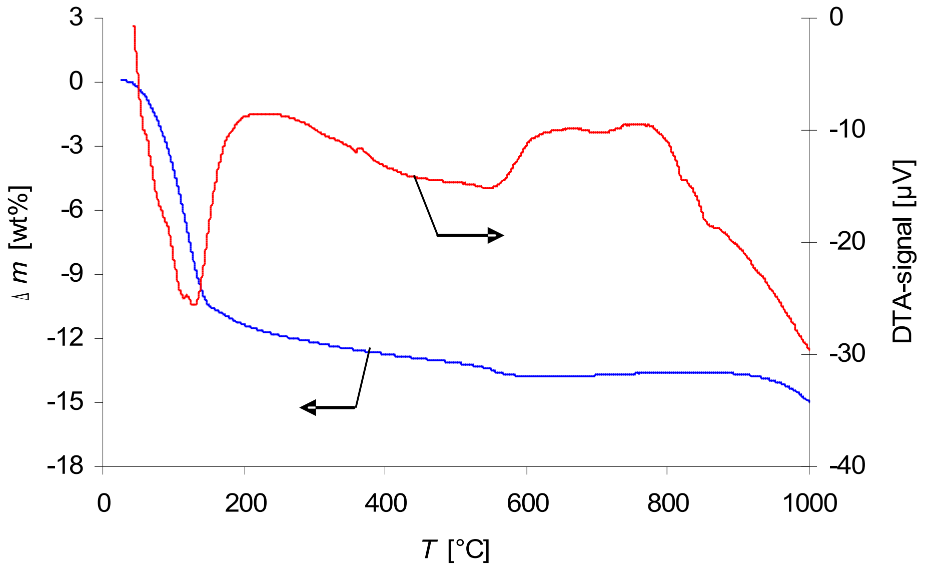

Figure S1: TGA and DTA curves of freshly prepared KF loaded alumina **SRS1a** (32 wt % KF; cf. Table 2 of the manuscript).

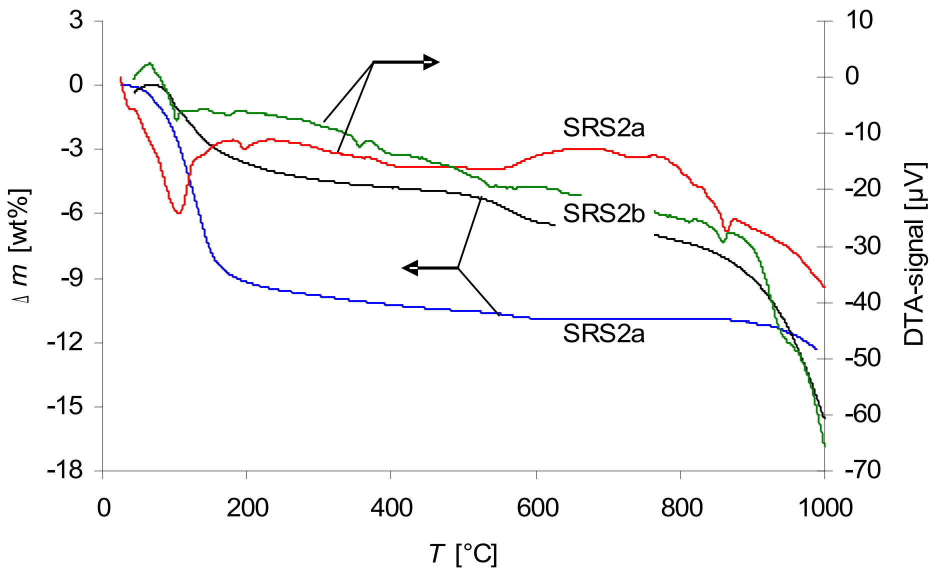

Figure S2: TGA and DTA-curves of freshly prepared KF-loaded alumina **SRS2a** (32 wt % KF; cf. Table 2 of the manuscript) and calcined material **SRS2b** (32 wt % KF, 2 h at 300 °C; cf. Table 5 of the manuscript).

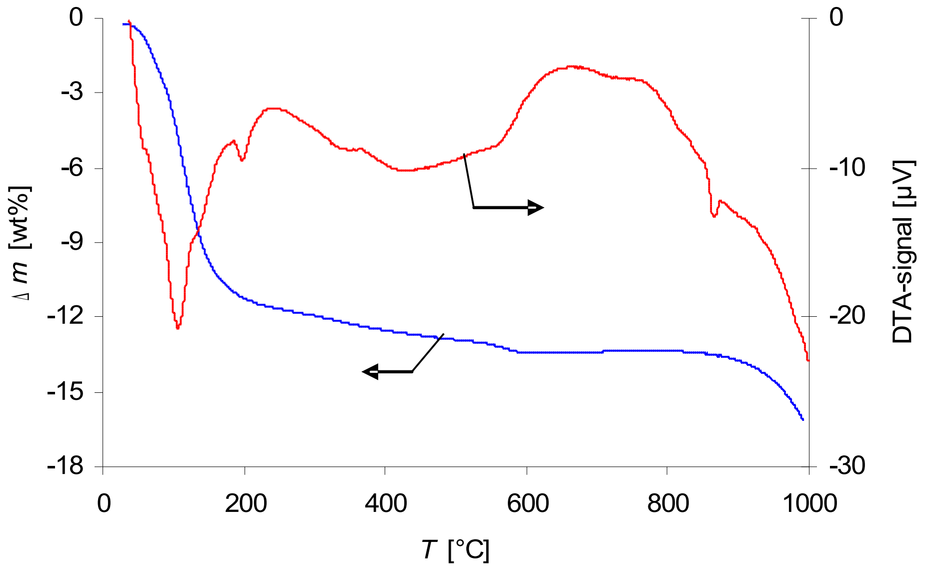

Figure S3: TGA and DTA-curves of freshly prepared KF-loaded alumina **SRS3a** (32 wt % KF; cf. Table 2 of the manuscript).

TGA curves for the freshly prepared KF loaded aluminas **SRS1a–3a** (Figures S1–3) reveals intensive weight loss in the range of 50–150 °C, which is accompanied by an intensive exothermic signal in the DTA curves. The signal combinations are typical for the loss of weakly bound material. The absolute weight loss was in the same region as shown for the

water content measured by Karl–Fischer-titration (cf. manuscript text). In light of this and the absence of any further low-boiling material in the as-prepared materials, it is assumed that the material loss was due to dehydration of physisorbed water. The release of chemically bound water takes place at higher temperatures and is accompanied by a change in phase composition of the aluminas, which was not observed.

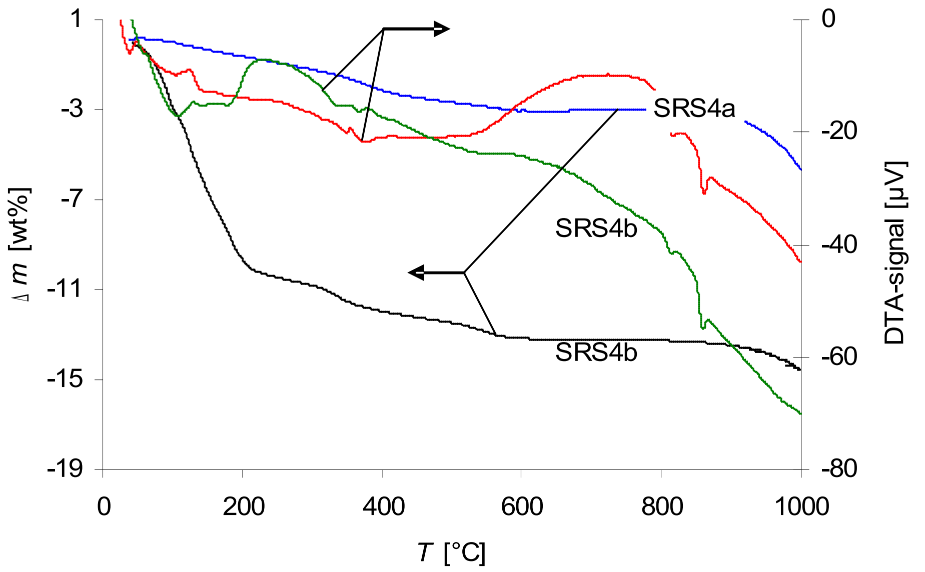

Figure S4: TGA and DTA-curves of commercially available KF-loaded alumina **SRS4a** (32 wt % KF) and hydrated material **SRS4b** (32 wt % KF; cf. Table 5 of the manuscript and experimental part).

Water release for the commercially available material **SRS4a** (Figure S4) is much lower, indicating a significantly lower water content than observed for the freshly prepared materials. Calcination of self-prepared **SRS2a** for 2 h at 300 °C resulted in material **SRS2b** showing a strongly decreased water release (cf. Figure S2). The reverse reaction – hydration of **SRS4a** leading to **SRS4b** – resulted in a material with increased water content (Figure S4), accompanied by increased activity in the Suzuki–Miyaura model reaction (cf. Table 5 of the manuscript).

XRD-measurements of the freshly prepared KF loaded aluminas

X-ray diffraction (XRD) experiments were performed on a Kristalloflex D5000 diffractometer (Siemens, Germany) with CuK$_\alpha$ radiation (E = 37 keV; I = 40 mA) at room temperature using a Ni filter to identify the crystalline phases of the samples.

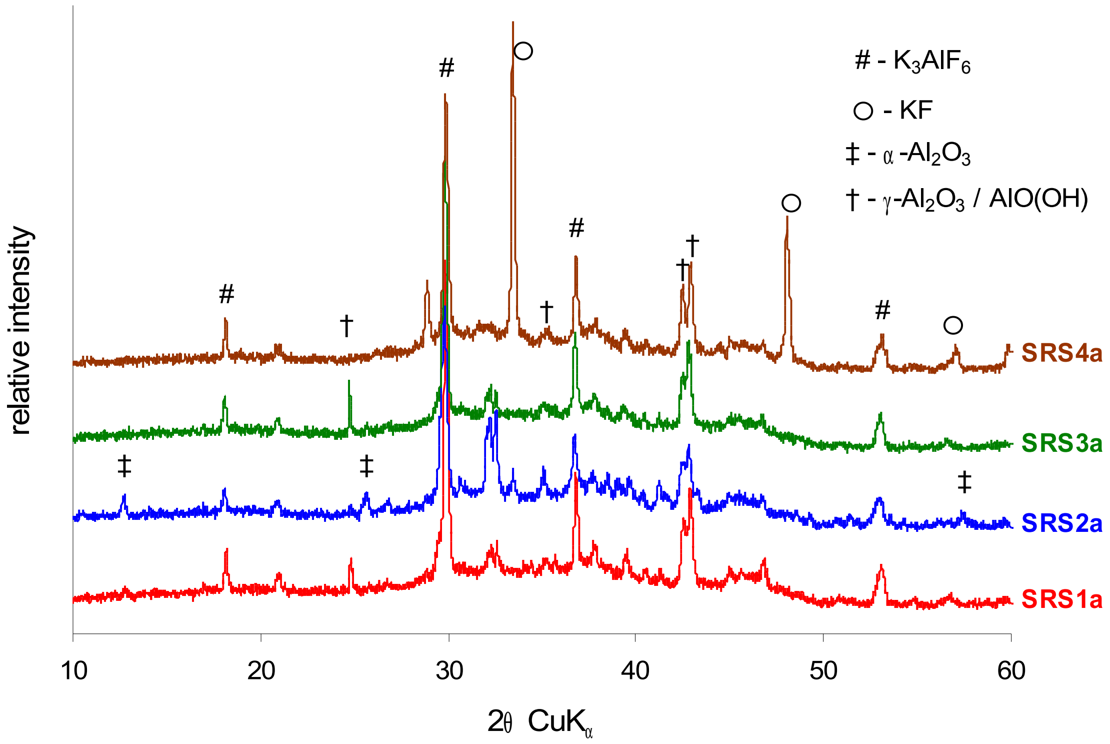

Figure S5: XRD spectra of the as-prepared KF loaded aluminas (**SRS1a–3a**; 32 wt % KF) and of commercially available **SRS4a** (as-received, 32 wt % KF).

Acknowledgements

The assistance of B. Fähndrich (Institute for Technical Chemistry and Environmental Chemistry, Jena; thermal analyses) and of Dr. M. Müller (Otto-Schott Institute for Glass Chemistry, Jena; XRD-analyses) is gratefully acknowledged.
